# The Effects of COVID-19 on the Emotional and Social Stability, Motivation and Attitudes of Gifted and Non-Gifted Children in Greece

**DOI:** 10.3390/children10040706

**Published:** 2023-04-10

**Authors:** Alexandros Papandreou, Alkistis Mavrogalou, Aristodemos-Theodoros Periferakis, Argyrios Periferakis, Ioana Anca Badarau, Ovidiu Popa-Velea, Cristian Scheau

**Affiliations:** 1 Elkyda, Research & Education Centre of Charismatheia, 17675 Athens, Greece; 2 Department of Physiology, The “Carol Davila” University of Medicine and Pharmacy, 050474 Bucharest, Romania; 3 Akadimia of Ancient Greek and Traditional Chinese Medicine, 16675 Athens, Greece; 4 Department of Medical Psychology, Faculty of Medicine, University of Medicine and Pharmacy “Carol Davila”, 050474 Bucharest, Romania

**Keywords:** gifted children, home confinement, distance learning, emotional and social stability needs, motivation, attitudes, COVID-19

## Abstract

Gifted children exhibit advanced cognitive abilities, usually beyond their emotional development, which puts them at higher risk of the negative consequences of isolation. This study investigates the effects of distance learning and home confinement on the emotional and social stability, motivation, and attitudes of gifted and non-gifted children in Greece. Our study includes two subsets, from before (September 2017 to March 2020) and after the outbreak of the COVID-19 pandemic (April 2020 to March 2022). The analysis indicated that home confinement and distance learning caused children to create a stronger attachment with their parents, and it increased the involvement of parents in their child’s school experience. Non-gifted children displayed high levels of certain attitudes (perfectionism, desire for acceptance, and condescending behavior) and demonstrated elevated motivation. Gifted children in the pre-COVID-19 period had already displayed increased levels of condescending attitude, which is assumed to be the result of already existing expectations from their parents. The pandemic further increased this attitude, as a result of the higher expectations of their parents. Overall, the study highlighted the importance of children to have more than one support system and the need for them to strengthen their self-image.

## 1. Introduction

There is no single widely accepted definition of giftedness. Standardized intelligence quotient (IQ) tests remain the most dominant way in which gifted individuals are generally identified. However, they are not fully adequate for a comprehensive and diagnostically appropriate identification of these individuals, as they do not always take into consideration the most relevant qualitative characteristics associated with giftedness. Several examples in this respect include creativity, leadership skills, artistic expression, perceptual ability, emotional stability [1,2], as well as an internal locus of control [3]. Significant methodological difficulties in exploring gifted children stem from the great variations in the characteristics present in a single individual, and the age at which individuals exhibit their giftedness [4]. Further difficulties are caused by the fact that gifted children may display an asynchrony between their cognitive, emotional, and physical development [5].

As a whole, gifted children typically exhibit advanced cognitive abilities that can surpass their emotional and/or physical development [6,7]. Consequently, their advanced cognitive abilities may hinder their social skills and peer relationships. Their increased self-awareness and deviation in their interests and performance may negatively influence their relationship with their classmates [6]. Eventually, gifted children may end up feeling lonely and marginalized, which sometimes results in a condescending attitude, as a compensatory mechanism. Still, in other occurrences, they may conform with the norms of their social group [8]. These individuals generally score higher on intrinsic motivation [9,10] and may have a greater sense of self-concept, compared to their peers [8].

In terms of academic life, gifted students may perceive courses within their curriculum to be unchallenging, slow-paced, and repetitive, with few opportunities to use their own initiative and critical thinking strategies [11], and they may feel an overall lack of attention being paid to their educational needs [12]. Acceleration, differentiation, full-time separate schools, and special classrooms have all been proposed to better adjust the educational process to the needs, interests, and motivations of gifted students [12,13]. Still, this does not always impede the self-perception of underachievement and disengagement [14]. 

In the last two decades, gifted children in Greece have been included, from a legal point of view, in the “special educational needs” category [15]. However, this has not been associated with any special adjustment/enrichment to the general curriculum of the Greek educational system, or sustainable specialized programs to acknowledge the specific needs of gifted children. 

In the context of the restrictions brought on by the COVID-19 pandemic, remote learning has been used as a tool for maintaining the curriculum’s progress rate and offering a substitute for socializing. A large body of research has investigated these effects, with ambivalent results. For example, online teaching has been perceived by medical university students as being advantageous, in terms of time management and seeking information [16], increased flexibility and productivity [17], and improvement in media-related competence [18]. Still, other authors suggested that the academic motivation of children exposed to online teaching has decreased, especially younger children [19], while also noting poor engagement [20] and a decline in performance [21].

These effects have also been investigated in gifted children, usually indirectly, through parental questionnaires and interviews [22,23]. When investigated from a child’s perspective, gifted students reported that remote learning was not appropriate and efficient, and they generally expressed a negative attitude towards online teaching [24]. These children also exhibited a series of negative outcomes, such as sleep disorders, a lack of motivation, laziness, and boredom [25]. Compared to community samples, the differences seem to be aggregated around cognitive aspects, and less or not at all around emotional ones [26]; although, in many of the aforementioned studies, there was no baseline measurement of their pre-COVID-19 state and its potential influence on the different populations.

During the pandemic, the participation of parents in the education of their children increased, with many reports from parents indicating that they believed they had to substitute the teacher during the period of online learning [27]. Overall, the pandemic may have affected the relationship of the children with their parents and teachers, with this effect being evident from all sides [28]. 

This study examined the effects of distance learning and home confinement due to COVID-19 on the emotional and social stability, motivation, and attitudes of gifted and non-gifted children in Greece. We have found no previous studies that have investigated these effects of the pandemic in Greece. Furthermore, there are quite a few previous studies which investigate the pre/post pandemic differences between the study variables from the perspective of gifted and non-gifted children, whose development and external environments are different. The results are also going to be discussed in terms of the parent–child relationship deviation that took place. More specifically, the dimensions investigated were regarding (1) “Emotional and social stability”—emotional balance and perception of a strong family support system, (2) “Motivation”—the dominant source of motivation (internal, external), or the lack of motivation, and (3) “Attitudes”—conforming and condescending attitudes, desire for acceptance, freedom of choice, and perfectionism.

## 2. Materials and Methods

### 2.1. Design

To better capture the changes that occurred in the variables, the study included a mixed quantitative and qualitative analysis run at two different times, namely before the COVID-19 pandemic (September 2017 to March 2020) and after its beginning (April 2020 to March 2022).

### 2.2. Participants

The study was based on a convenient sample of children who approached “Elkyda”, the Educational Research Centre of Charismatheia (https://charismatheia.edu.gr/program/elkida/, accessed on 11 February 2023), to take part in Elkyda’s programs. Elkyda’s primary aim is advancing research for the remodeling of education. The scope of research extends into the scientific areas related to the design of differentiated education programs, design and evaluation of innovative learning objects, and through the exploration of educational attitudes of specific categories, such as gifted children. In this center, psychometry is constantly used, with an emphasis on the assessment of competencies that weigh critical thinking, creativity, and cultivation of skills. The use of technology (specifically digital tools) in providing education is supported as well. 

The Centre offers extracurricular programs to support gifted children. In order for children to take part in the “master classes”, they need to first undertake a holistic evaluation of themselves, including questionnaires given to the child and separately to the parent, a semi-structured interview, and a test-IQ. All these are performed in order to make sure that each child can gain something meaningful and significant from the program. At the beginning of this evaluation, an informed consent agreement is given to the parents, who can decide whether the quantitative results of the questionnaires can be used for research purposes in the future, in an anonymized way and following ethical guidelines. 

Data from 958 elementary school children (550 boys, 408 girls, mean age 9.34 ± 1.74) from approximately 400 different schools across Greece were used in the study. The majority of participating schools were located in Athens and included both private and public facilities. These data include questionnaire answers from September 2017 to March 2022. For the purposes of this study, the sample was divided into two groups: pre-COVID-19 (using data from September 2017 to March 2020) and post-COVID-19 (using data from April 2020 to March 2022). In total, the sample was separated into four groups: pre-COVID-19 and gifted, pre-COVID-19 and non-gifted, post-COVID-19 and gifted, and post-COVID-19 and non-gifted (Table 1).

### 2.3. Inclusion and Exclusion Criteria

The children were identified as gifted based on a quantitative measure of their perceptual ability (using the IQ Figure Reasoning Test, FRT [29]) and on a qualitative measure of their overall behavior and stability (using the semi-structured interview). This evaluation was based on the three-ring conception of giftedness, which defines it as encompassing “three inter-related components: above-average ability, high levels of task commitment and high levels of creativity” [30]. Children who were not identified as gifted were included in the non-gifted groups.

### 2.4. Ethics

All the procedures, investigations, and actions within the study were carried out following the rules of the Declaration of Helsinki of 1975, revised in 2013 [31]. Informed consent was obtained from all participants and their legal guardians (parents) before they participated in the study. The anonymity of all subjects was maintained throughout the study and afterwards. Written informed consent for publication was obtained from all study participants.

### 2.5. Instruments

The selection of the study instruments was based on the provision of an accurate estimation of the children’s developmental stage (i.e., whether they have advanced cognitive abilities in comparison to other children of the same age), a comprehensive understanding of their behavior and attitudes in different environmental settings, as well as a good assessment of their competence level. These instruments included an IQ test, a set of questionnaires administered to the child, and, separately, to the parents. These instruments have been consistently used since 2012 in Elkyda, and have been internally standardized and validated in the Greek population of children between the ages of 6 and 16.

1. The IQ-test was represented by the two forms of the FRT [29]. Children from 6 to 10 years old were administered the FRT Type J (FRT-J), which consisted of 2 example questions and 25 test questions. Children who were 11 years old and above took the FRT Type B (FRT-B), which consisted of 2 example questions and 45 test questions. Each item included a 3 × 3 matrix of geometric structures, with the lower right structure missing. The children had to understand the pattern that the geometric structures formed and choose, from a selection of answers, the answer that completed the pattern. The children were fully supervised during this process and the test was time constrained. This test has been previously assessed for reliability and validity. In order to classify a child as gifted or non-gifted, a series of specific thresholds were taken into consideration according to the test manual, and depending on the number of correct answers and the age of the child. The children were then clustered into 5 distinct groups (1—lower than average developmental stage, 2—average developmental stage, 3—above-average developmental stage, 4—higher developmental stage, 5—highest developmental stage). The threshold for all clusters depended on the distance of a given score from the normally distributed mean of the scores. Children belonging to groups 4 and 5 were taken into consideration for this study as being gifted.

2. The children had to answer a series of questions, designed in the form of a section from a gamification platform. The questionnaire included multiple questions. For the purposes of this study, only questions concerning the emotional and social stability, the motivation, and attitude of children were analyzed. The theoretical framework that forms the basis of these constructs and the way we perceive the emotional and social stability of children is the Social Cognitive Theory [32]. Additionally, the Goal Orientation Theory was used as an additional framework for the questions around motivation, since it is more specific to children and education, and it suggested reasons for engaging in achievement behavior and the way children judge failure and success [33]. The questions were in the form of distinct statements and the children had to choose the degree at which a certain statement applied to them, on a Likert scale from 1–5 (“It never applies to me”, “It sometimes applies to me”, “It is both valid and invalid to me”, “It applies most of the times to me” and “It always applies to me”). The participants were able to omit a question that did not apply to them and/or that they could not decide how to answer. To increase comfort, the children were aware that their answers would not be seen by their parents. Throughout the questionnaires, many questions were repeated in a positive or negative form, within the same or in a different questionnaire. In the case of a negative form of a specific question, the answer was reversed (by subtracting 6—the answer). This repetition acts as a validity indicator to understand whether children were able to provide consistency and accuracy in their answers. If answers to two similar questions had a distance equal to or greater than two units, then this was flagged as an inconsistency. Flagged questions were omitted from the data analysis. 

For example, for the metric “Parents are supportive”, among other questions, the questions “My parents encourage me to answer questions even if I make mistakes” and “My parents do not like it when I make mistakes” are both included in separate parts of the questionnaire. These questions are an example of a positive and negative form of statement, where the same thing is asked but in a different way to ensure validity. If a child answers 4 in the second negative statement, then this reversed is 2. If the child answers 5 in the first statement and 2 in the second statement (after it has been reversed), then this is flagged as a difference of 3 units. 

The internal consistency of the questionnaire was verified for each metric. We obtained Cronbach’s alpha values higher than 0.7 for one metric from each group (i.e., “Display Emotional Balance”, “Have Internal and External Motivators”, and “Display a Conforming Attitude”), while the metric “Lack Motivators” showed a value higher than 0.8. Moreover, the “Have Internal and Intrinsic Motivators”, “Display a Condescending Attitude”, and “Display Perfectionism” metrics achieved Cronbach’s alpha values higher than 0.6.

3. The parents had to answer a subset of these questionnaires provided to the children. They were asked to answer them from the perspective of the child, i.e., as their child would have answered these questions. Similarly to the previous test, if inconsistencies occurred in the answers provided by the child or the parent, they were flagged and omitted from the data analysis. Following the example in the child’s questionnaire, the parents were asked “My parents do not like it when I make mistakes” and they had to reply from the perspective of their child in order to have an external validation of the answers from the children. If the parent’s answers to the questions had a distance equal to or greater than 2, then this was flagged as an inconsistency, and it was omitted from data analysis.

### 2.6. Data Analysis

The calculation of the metrics used for the evaluation of children was based on the quantitative answers from the questionnaires, and on the qualitative cross-examination of the questionnaires with the interview. The total number of questions was 296, corresponding to 11 metrics. Their values were normalized between 0 and 100.

#### Statistical Analysis

Descriptive statistics were measured using the mathematic equations of Excel. Additionally, a two-tailed unequal variance *t*-test was performed, using Excel’s statistical tests, on the values of each metric between pre- and post-COVID-19 groups, as well as between gifted and non-gifted children. Considering the main aim of the study, to emphasize the effects of distance learning and home confinement on children, a focus was made on the differences between pre- and post-COVID-19 groups, and not on the difference between gifted and non-gifted children. Cronbach’s alpha was used as a reliability coefficient in order to measure the internal consistency of each metric.

## 3. Results

To investigate whether there was a change in the attitudes and behaviors of gifted and non-gifted children during the COVID-19 pandemic, the average values of metrics exploring the social and emotional stability, the attitudes and the motivation of gifted and non-gifted children were calculated.

**Figure 1:**
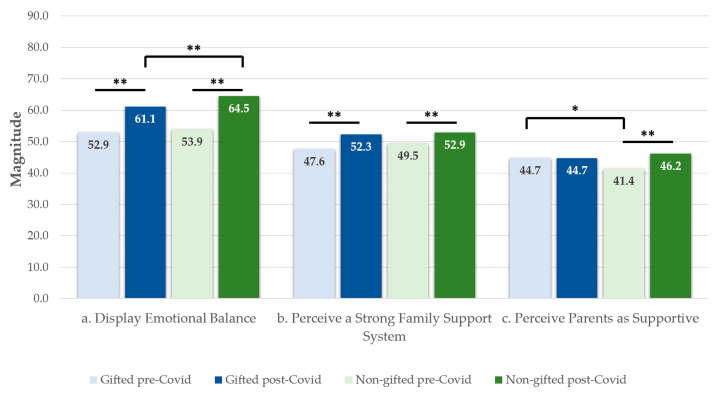
Pre- and post-COVID-19 social and emotional stability in gifted and non-gifted children (Student’s *t*-test, * *p* < 0.05, ** *p* < 0.01). The three metrics are represented in the figure. Bars represent values within each study group.

In the metric “Display Emotional Balance”, there was a significant (*p* < 0.01) post-COVID-19 increase in both gifted and non-gifted children (Figure 1a). Non-gifted children reported a significantly (*p* < 0.01) higher emotional balance. Furthermore, the metric “Perceive a strong Family Support System” changed in both groups (Figure 1b; *p* < 0.01), indicating a shift in the attitude and behavior of parents towards their children. Non-gifted children reported that their family support system was strengthened in the post-COVID-19 period to a greater degree compared to gifted children. Interestingly, in the metric “Perceive Parents as Supportive” (Figure 1c), only non-gifted children indicated a significant (*p* < 0.01) increase, whereas there was no change in gifted children, who reported an overall lower average value (Supplementary Table S1).

**Figure 2:**
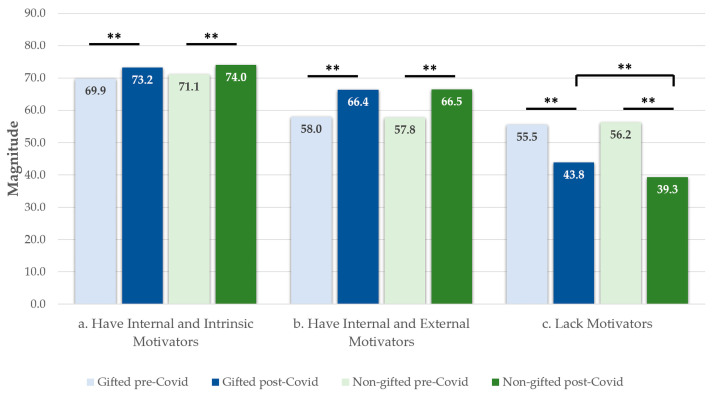
Pre- and post-COVID-19 types of motivators in gifted and non-gifted children (Student’s *t*-test, ** *p* < 0.01). The three metrics are represented in the figure. Bars represent values within each study group.

There was a significant increase from pre- to post-COVID-19 in both gifted and non-gifted children in both “Have Internal and Intrinsic Motivators” and “Have Internal and External Motivators” (*p* < 0.01; Figure 2a,b). Furthermore, the metric “Lack Motivators” significantly (*p* < 0.01) dropped post-COVID-19 in both groups (Figure 2c) (Supplementary Table S2).

**Figure 3:**
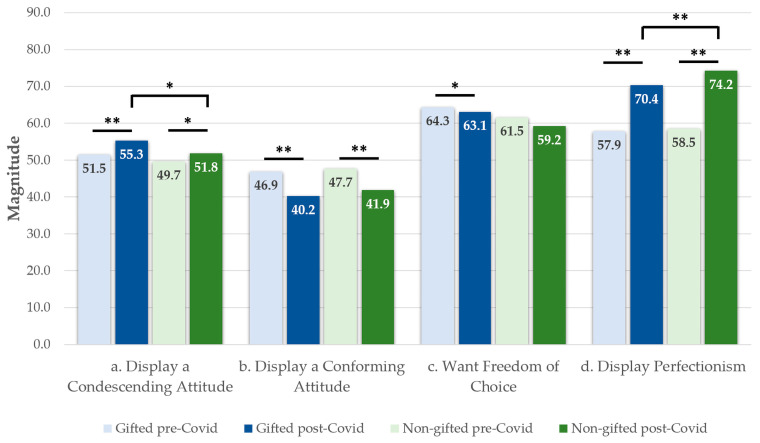
Pre- and post-COVID-19 attitudes in gifted and non-gifted children. (Student’s *t*-test, * *p* < 0.05, ** *p* < 0.01). The four metrics are represented in the figure. Bars represent values within each study group.

The average values of metrics exploring attitude in both gifted and non-gifted children are illustrated in Figure 3. There was a statistically significant increase in the condescending attitude of both gifted (*p* < 0.01) and non-gifted (*p* < 0.05) children (Figure 3a). There was also a statistically (*p* < 0.01) significant decrease in the conforming attitude of both groups (Figure 3b). Even though in the pre-COVID-19 period, the average values of the condescending attitude had been higher than those of the conforming attitude, a further increase in the condescending attitude has been observed in the post-COVID-19 period. This complies with the observed post-COVID-19 decrease in the conforming attitude. Additionally, both gifted and non-gifted children had a significantly greater desire for acceptance from external factors in the post-COVID-19 period (*p* < 0.01; Figure 3c). This effect was significantly (*p* < 0.05) higher in non-gifted children. In the metric “Want Freedom of Choice”, there was a significant (*p* < 0.05) decrease in non-gifted children and an insignificant decrease in gifted children (Figure 3d). Finally, perfectionism was significantly higher in the post-COVID-19 period in both gifted and non-gifted children (*p* < 0.01) (Supplementary Table S3).

All of the 10 metrics mentioned above have also been studied regarding the relationship to the subject’s gender. The complete results and associated analysis of the data are presented in Supplementary Table S4. We found that post-COVID-19, gifted boys scored higher than girls in the metrics “Lack motivators” (*p* < 0.01) and “Display a Conforming Attitude” (*p* < 0.05) while scoring lower in the metric “Display perfectionism” (*p* < 0.05). There were no gender-based differences in gifted children in the pre-COVID-19 population. Moreover, in non-gifted children, post-COVID-19, boys scored higher in the metrics “Lack motivators” (*p* < 0.01) and “Display a Condescending Attitude” (*p* < 0.01), while scoring lower in “Perceive a Strong Family Support System” (*p* < 0.01) and “Display perfectionism” (*p* < 0.01).

## 4. Discussion

In this study, differences regarding the gifted and non-gifted children’s emotional and social stability, motivation, and attitudes were compared, before and after the outbreak of the COVID-19 pandemic. The specifics of our study population (i.e., gifted vs. non-gifted children enrolled in the Greek educational system) and the uniqueness of the COVID-19 pandemic and associated consequences compelled us to use a more adequate instrument specifically designed for the assessment of the required metrics. The results are going to be discussed in terms of the parent–child relationship deviation that took place.

### 4.1. Emotional and Social Stability

The changes observed in the metrics “Family Support System” and “Parents are compassionate” indicated that both gifted and non-gifted children experienced a shift in the relationship with their parents, as a result of home confinement and distance learning (Figure 1b,c). It is possible that as a result of distance learning, parents may have become more aware of the school experience of their children and their level of participation, and thus formed a better image of their children within the classroom. Previous studies regarding parental involvement have indicated that parents could easily engage in attitudes where they antagonize the teachers work if they perceive that the curriculum does not fit their standards [34,35]. Therefore, parents may have increased their involvement in their children’s online learning [36,37], and consequently their expectations from their children. Additionally, due to home confinement, parents became the main and most available support for their children (in line with [36]). This likely created a shift to stronger dependencies of children towards their parents (in line with [38]). In turn, children felt more secure in a confined environment, and the stronger attachment style and security received from parents may have been interpreted as stronger emotional balance (Figure 1a). As a result of the direct absence of the other support systems (school and friends), the children based their emotional stability on an insubstantial and temporary environment in which they felt safe. This one-dimensional target was less complicated for children, who focused on fulfilling the demands of their main support system, their parents, without having other interfering factors such as their friends’ perceptions. 

Even though it appears that home confinement and distance learning positively affected children, it is assumed that children became more prone to emotional manipulation. As children found their emotional stability in a one-dimensional source of support, this brings the risk of decreasing social and emotional stability in the long-term.

In non-gifted children, the observed involvement was interpreted as increased interest from their parents, so they overall perceived it as positive (Figure 1b). Conversely, in the most gifted children, it is presumed that parental overinvolvement had already been present in the pre-COVID-19 period; therefore, they may have not perceived their parents as necessarily more supportive, but as exerting some degree of emotional pressure on them. 

### 4.2. Motivation

Both gifted and non-gifted children reported increased levels of “Internal and Intrinsic Motivators” and “Internal and External Motivators” (Figure 2a,b, respectively). The difference between these two metrics indicates the amount of external motivation that children experienced. As displayed in Figure 1a,b, external motivators increased, indicating that children were more prone to be affected by them. It is possible that gifted and non-gifted children interpreted the increased involvement of their parents as external motivators, even to the point where they internalized some of them. Given that there was only one major source of external expectation during the pandemic, a small decrease in the demotivators was present, since children had no conflicting targets and only one clear goal in order to obtain acceptance: to fulfill their parents’ expectations (Figure 2c). 

### 4.3. Attitudes

Given that children had a single and clear target to fulfil their parents’ wishes, it was expected that there would be an increase in the reports of condescending attitude and a decrease in the conforming attitude (as observed in Figure 3a,b). Regarding the conforming attitude, the post-COVID-19 values were significantly lower in girls compared to boys. Children were not located in a school environment where they could interact with their peers, and thus created stronger dependencies on their parents. This effect, along with the lack of self-acceptance, heightened the condescending attitude of children, in order to meet the new expectations of their parents and achieve external acceptance. Non-gifted children reported lower levels of condescending attitude compared to gifted children. On the other hand, gifted children already had increased expectations from their parents, as indicated by the increased levels of condescending pre-COVID-19 attitudes. However, as a result of the new parent–child relationship, the condescending attitude of gifted children was also heightened, in an effort to meet the new expectations. Possibly, this new condescending attitude does not illustrate a permanent characteristic, and is not fully adopted by children since it takes a couple of years for such a transition to take place [39,40]. Nevertheless, such behaviors can become permanent if they are not addressed on time, since they act as structural elements on which children can build their identity [41]. 

The shift in the parent–child relationship also likely resulted in children restraining their freedom and consequently their self-concept (Figure 3c). Gifted children, who generally hold higher levels of intrinsic motivation [10], still wanted to define themselves and have freedom of choice (Figure 3c). Gifted children were also used to the increased involvement of their parents, and thus did not experience a completely new situation that could restrain their freedom in a significant way. 

Perfectionism was significantly higher in the post-COVID-19 period (Figure 3d). To some extent, this metric includes the fear of failure that children may experience towards the external expectations. In addition, the metric revealed statistically significant higher values in girls compared to boys. Given that the main source of the external expectations was solely represented by the parents during COVID-19 (one-dimensional source of support and demands), a small decrease in the demotivators was present, since children focused on a clear goal to fulfil their expectations to obtain acceptance. However, this was more expressed in girls rather than boys in our study population. The extremely low levels of demotivators reported by non-gifted children are compelling, with the increased desire for acceptance and less expressed freedom of choice. This is in line with studies that have demonstrated that parental criticism and high expectations have been found to be predictors of negative perfectionism in gifted children [42,43]. Additionally, it has been suggested that parental overinvolvement could hinder academic motivation and achievement [44,45]. As mentioned before, the one-dimensional source of support may have oriented the behavior towards fulfilling the demands of the parents, resulting in perfectionism as a response mechanism to both external and internalized pressures.

Both gifted and non-gifted children indicated a tendency to improve their academic performance, possibly as a result of home confinement and distance learning and closer parental supervision. This was supported by the increased levels of perfectionism. Even though this effect seems to occur in both gifted and non-gifted children, non-gifted children do not express the pressure they feel to achieve an increase in their performance, and do not face the fear of a possible failure in this. It could be hypothesized that non-gifted children do not base their identity on their performance. In contrast, gifted children derive their identity from their performance, and the ability to succeed and to meet the expectations of people around them [46]. Thus, pressures from their parents are reflected by the high values in the children’s lack of motivation and fear of failure. In conclusion, as a result of home confinement and distance learning due to the pandemic, non-gifted children did not have a change in their attitude and behavior. On the other hand, gifted children felt more pressure to maintain this high image of performance and achievement, causing a further increase in their perfectionism. Therefore, we can consider that perfectionism is not an inherent characteristic but rather a result of pressures, comparison with other children, and a low level of demands. Thus, it is a result of the environment rather than the actual abilities of the child.

### 4.4. Limitations

Our study has several limitations. One strong limitation is the lack of validation of our questionnaire, which was specifically designed to address the study population in the context of the Greek educational system. We obtained acceptable internal consistency values for 4 metrics, while another 3 yielded values higher than 0.6. Future studies are required to improve the design of the questionnaire through factor analysis and to ensure good internal consistencies in all metrics, as well as to validate the questionnaire for this population. The assessments were performed at different time points before and after the beginning of the COVID-19 pandemic; therefore, both children and their parents might have been biased by various social, economic, or personal events in this unstable time period. Additionally, given that there were different periods during the pandemic in which these assessments took place, the children’s distance learning status may have been different. In addition, it should be noted that this paper describes a trend exhibited in the attitudes and behaviors of gifted and non-gifted children during the pandemic. Certainly, not all children displayed these behaviors, and each child expressed them to a different degree, depending on their situation and home environment. To this end, the intention of this study is not to find a ground rule, but to provide insight, contributing to the decoding of the mechanics and forces that drive children’s behaviors and attitudes towards their parents and peers. 

## 5. Conclusions

This study highlights the low adequacy of the Greek educational environment to fulfill the needs and interests of children, as well as the standards of most parents, especially during the pandemic. The study was designed on the framework of the Social Cognitive Theory and Goal Orientation Theory. We uncovered gender-based differences in several metrics measured in gifted children post-COVID-19, which might reveal intrinsic differences accentuated by the stress induced by home confinement and associated lifestyle changes. Parents overstepped the role of the teacher and became overinvolved with the children’s performance within the school. This created emotional pressure for the children, who correlated the acceptance they received from their parents with their performance. This paper stresses the need to change the children’s relationships with their family, school, and friend support systems, and maintain their multi-dimensional social and emotional stability. Better communication between the family and school could result in a healthier expression of parental expectations via the teachers. Therefore, children could achieve higher acceptance in the family and a better self-image, regardless of their performance at school, as their identity will not be solely defined by their ability to succeed. We believe that this study is a good reflection of the particular conditions that gifted children are facing in the Greek educational system, and can serve as ground work to develop an integrative approach in the management of these children.

## Figures and Tables

**Table 1:** Participants in the study.

Children	Pre-COVID-19	Post-COVID-19
Gifted	87	147
Non-gifted	87	637

## Data Availability

The data presented in this study are available on reasonable request from the corresponding authors.

## Supplementary Materials

The following supporting information can be downloaded at https://www.mdpi.com/article/10.3390/children10040706/s1, Supplementary Table S1. Pre- and post-COVID-19 social and emotional stability in gifted and non-gifted children. Supplementary Table S2. Pre- and post-COVID-19 types of motivators in gifted and non-gifted children. Supplementary Table S3. Pre- and post-COVID-19 attitudes in gifted and non-gifted children. Supplementary Table S4. Magnitudes of the metrics measured in our study stratified by gender.
