# Supplementary material for: The Effects of COVID-19 on the Emotional and Social Stability, Motivation and Attitudes of Gifted and Non-Gifted Children in Greece
Source: Children (Basel). 2023 Apr 10;10(4):706. doi: 10.3390/children10040706 (PMC10137062; doi:10.3390/children10040706)
Supplement: Supplementary file 1 [file children-10-00706-s001.zip › children-2247515-supplementary.pdf]

Table S1. Pre- and post-COVID-19 social and emotional stability in gifted and non-gifted children.

**Display Emotional Balance**				
		N	Mean	SD
Gifted	pre-COVID-19	86	52.9	10.5
	post-COVID-19	145	61.1	8.3
Non-gifted	pre-COVID-19	86	53.9	9.3
	post-COVID-19	612	64.5	7.0
**Perceive a Strong Family Support System**				
Gifted	pre-COVID-19	78	47.6	8.4
	post-COVID-19	143	52.3	5.1
Non-gifted	pre-COVID-19	83	49.5	6.0
	post-COVID-19	567	52.9	5.5
**Perceive Parents as Supportive**				
Gifted	pre-COVID-19	63	44.7	3.4
	post-COVID-19	139	44.7	7.5
Non-gifted	pre-COVID-19	78	41.4	5.3
	post-COVID-19	553	46.2	6.8

(N = total number, M = mean, SD = standard deviation)

Table S2. Pre- and post-COVID-19 types of motivators in gifted and non-gifted children.

**Have internal and intrinsic motivators**				
		N	Mean	SD
Gifted	pre-COVID-19	86	69.9	9.6
	post-COVID-19	145	73.2	6.1
Non-gifted	pre-COVID-19	87	71.1	7.5
	post-COVID-19	610	74.0	8.4
**Have internal and external motivators**				
Gifted	pre-COVID-19	87	58.0	7.6
	post-COVID-19	144	66.4	6.1
Non-gifted	pre-COVID-19	87	57.8	7.1
	post-COVID-19	613	66.5	6.8
**Lack motivators**				
Gifted	pre-COVID-19	87	55.5	12.8
	post-COVID-19	144	43.8	10.9
Non-gifted	pre-COVID-19	87	56.2	12.8
	post-COVID-19	595	39.3	11.9

(N = total number, M = mean, SD = standard deviation)

Table S3. Pre- and post-COVID-19 attitudes in gifted and non-gifted children.

**Display a Condescending Attitude**				
		N	Mean	SD
Gifted	pre-COVID-19	86	51.5	5.6
	post-COVID-19	145	55.3	6.2
Non-gifted	pre-COVID-19	87	49.7	5.6
	post-COVID-19	627	51.8	7.0
**Display a Conforming Attitude**				
Gifted	pre-COVID-19	86	46.9	7.6
	post-COVID-19	145	40.2	7.3
Non-gifted	pre-COVID-19	86	47.7	6.1
	post-COVID-19	618	41.9	6.7
**Want Freedom of Choice**				
Gifted	pre-COVID-19	73	64.3	11.6
	post-COVID-19	140	63.1	8.0
Non-gifted	pre-COVID-19	79	61.5	9.6
	post-COVID-19	563	59.2	10.2
**Display perfectionism**				
Gifted	pre-COVID-19	86	57.9	12.3
	post-COVID-19	143	70.4	12.2
Non-gifted	pre-COVID-19	87	58.5	11.1
	post-COVID-19	606	74.2	12.2

(N = total number, M = mean, SD = standard deviation)

Table S4. Magnitudes of the metrics measured in our study stratified by gender.

Metric	Non-gifted		Dif.	Gifted		Dif.
	Boys	Girls	(p value)	Boys	Girls	(p value)
Pre-COVID-19						
Display Emotional Balance	52.4 ± 11.4	56.4 ± 12.2	p=0.1284	52.6 ± 12.7	53.5 ± 17.3	p=0.7938
Perceive a Strong Family Support System	48.4 ± 9.0	51.2 ± 7.7	p=0.1582	47.0 ± 11.3	48.8 ± 11.0	p=0.5151
Perceive Parents as Supportive	41.4 ± 9.6	41.5 ± 9.9	p=0.9573	45.2 ± 9.6	43.8 ± 7.5	p=0.5481
Have internal and intrinsic motivators	56.9 ± 9.2	59.5 ± 9.7	p=0.2226	57.4 ± 12.3	59.1 ± 11.3	p=0.5515
Have internal and external motivators	69.4 ± 8.9	74.3 ± 8.0	**p=0.0135**	69.7 ± 9.5	70.2 ± 10.0	p=0.8033
Lack motivators	57.2 ± 15.4	54.4 ± 17.3	p=0.4401	57.1 ± 16.5	52.4 ± 19.4	p=0.2453
Display a Condescending Attitude	50.5 ± 7.1	48.2 ± 6.8	p=0.1580	51.1 ± 6.7	52.3 ± 7.6	p=0.4394
Display a Conforming Attitude	47.3 ± 7.2	48.5 ± 8.6	p=0.5044	47.5 ± 9.5	45.9 ± 9.6	p=0.4476
Want Freedom of Choice	62.8 ± 13.5	59.7 ± 16.0	p=0.3611	64.2 ± 14.9	64.6 ± 14.5	p=0.9093
Display perfectionism	56.6 ± 15.1	62.1 ± 18.6	p=0.1417	56.1 ± 17.4	61.4 ± 21.2	p=0.2154
Post-COVID-19						
Display Emotional Balance	64.3 ± 9.7	64.8 ± 9.2	p=0.5181	59.8 ± 10.5	62.9 ± 11.1	p=0.0946
Perceive a Strong Family Support System	51.8 ± 7.5	54.2 ± 8.4	**p=0.0003**	51.3 ± 7.8	53.8 ± 7.3	p=0.0550
Perceive Parents as Supportive	45.6 ± 10.3	46.9 ± 10.2	p=0.1467	44.3 ± 8.5	45.3 ± 9.4	p=0.5344
Have internal and intrinsic motivators	66.6 ± 10.3	66.3 ± 10.8	p=0.8132	65.7 ± 8.7	67.4 ± 9.6	p=0.2876
Have internal and external motivators	74.0 ± 9.0	74.0 ± 8.0	p=0.9960	72.4 ± 7.9	74.3 ± 7.2	p=0.1387
Lack motivators	41.6 ± 15.9	36.4 ± 15.4	**p=0.0001**	46.9 ± 14.8	39.5 ± 16.3	**p=0.0057**
Display a Condescending Attitude	52.7 ± 8.2	50.7 ± 9.2	**p=0.0043**	54.9 ± 6.9	55.8 ± 8.9	p=0.5229
Display a Conforming Attitude	41.3 ± 7.9	42.6 ± 9.0	p=0.0621	41.6 ± 9.0	38.3 ± 9.2	**p=0.0343**
Want Freedom of Choice	59.3 ± 14.0	59.0 ± 13.1	p=0.7774	62.0 ± 10.0	64.7 ± 10.7	p=0.1254
Display perfectionism	72.4 ± 15.2	76.4 ± 14.7	**p=0.0014**	68.2 ± 16.5	73.4 ± 13.1	**p=0.0469**

Dif. = difference between genders. Values represent the arithmetical mean ± standard deviation. The statistical difference between lots was calculated with independent samples t-test. Displayed p value is two-tailed and statistically significant p values are bolded in the table.
